# Genome Sequences of Two *Klebsiella pneumoniae* Isolates from Different Geographical Regions, Argentina (Strain JHCK1) and the United States (Strain VA360)

**DOI:** 10.1128/genomeA.00168-13

**Published:** 2013-05-02

**Authors:** Gary Xie, Maria S. Ramirez, Steven H. Marshall, Kristine M. Hujer, Chien-Chi Lo, Shannon Johnson, Po-E Li, Karen Davenport, Andrea Endimiani, Robert A. Bonomo, Marcelo E. Tolmasky, Patrick S. G. Chain

**Affiliations:** Genome Science Group, Bioscience Division, Los Alamos National Laboratory, Los Alamos, New Mexico, USAa;; Microbial Program, Joint Genome Institute, Walnut Creek, California, USAb;; Center for Applied Biotechnology Studies, Department of Biological Science, California State University Fullerton, Fullerton, California, USAc;; Instituto de Microbiologíay Parasitología Médica (IMPaM, UBA-CONICET), Facultad de Medicina, University of Buenos Aires, Buenos Aires, Argentinad;; Louis Stokes Cleveland Department of Veterans Affairs Medical Center, Cleveland, Ohio, USAe;; Department of Medicine, Case Western Reserve University School of Medicine, Cleveland, Ohio, USAf;; Institute of Infectious Diseases, Bern, Switzerlandg;; Departments of Pharmacology, Molecular Biology and Microbiology, Case Western Reserve University School of Medicine, Cleveland, Ohio, USAh

## Abstract

We report the sequences of two *Klebsiella pneumoniae* clinical isolates, strains JHCK1 and VA360, from a newborn with meningitis in Buenos Aires, Argentina, and from a tertiary care medical center in Cleveland, OH, respectively. Both isolates contain one chromosome and at least five plasmids; isolate VA360 contains the *Klebsiella pneumoniae* carbapenemase (KPC) gene.

## GENOME ANNOUNCEMENT

*Klebsiella pneumoniae* causes serious community- and hospital-acquired infections (1–7) and its virulence seems to be enhanced by its ability to acquire resistance to antibiotics, including carbapenems (3). Only a few virulence factors were identified, including a pathogenicity island in some strains (8–10). After nucleotide and restriction-map comparisons among several strains, a high heterogeneity zone was proposed (11–13).

*Klebsiella pneumoniae* strains JHCK1 (6, 13) and VA360 (13, 14) were draft sequenced to 539- and 751-fold coverage using Illumina GAIIx, resulting in 51,121,119 and 61,407,190 reads, respectively. We mapped 78% (JHCK1) and 91% (VA360) of the reads onto 30 *Klebsiella* replicons, including 9 chromosomes, 16 plasmids, and 5 phages, using the CLC aligner (CLCbio). *K. pneumoniae* NTUH-K2044 displayed the highest chromosome coverage at 91.21% with reads from JHCK1, while VA360 data covered 96.17% of the *K. pneumoniae* subsp. *pneumoniae* HS11286 chromosome. Additionally, five plasmids displayed >40% coverage using reads from both isolates. Three of the five plasmids are found in *K. pneumoniae* subsp. *pneumoniae* strain MGH 78578: pKPN3 (coverage with JHCK1/VA360 reads, 84.82%/77.39%), pKPN4 (79.41%/98.48%), and pKPN5 (48.77%/42.13%). The remaining two are *K. pneumoniae* subsp. *pneumoniae* HS11286 plasmids, pKPHS2 (69.36%/73.09%) and pKPHS5 (48.55%/43.33%). It had already been shown that strain JHCK1 harbors the 11-kb pKPN4-like plasmid pJHCMW1 (15). Therefore, preliminary analysis suggests that both *K. pneumoniae* JHCK1 and VA360 carry one chromosome and at least five plasmids.

*De novo* short-read assembly was also performed for each genome using IDBA-UD (16), resulting in 228 (97.17% of the reads) and 210 (96.54%) contigs >500 bp for JHCK1 and VA360, respectively. The draft genomes of JHCK1 and VA360 consist of 6,016,101-bp and 5,578,970-bp sequences, respectively, with an average G+C content of 57.5% for both. These two genome sequences were annotated using an in-house implementation of the Ergatis annotation pipeline (17).

There are 5,812 and 5,414 predicted protein coding genes within the genomes of *K. pneumoniae* isolates JHCK1 and VA360. Of these, 20% and 18% of the protein coding genes are annotated as hypothetical or conserved hypothetical proteins. Of those with functional predictions, 40 and 22 are associated with phages/prophages, while 129 and 103 are associated with resistance to antibiotics or toxin production. A *bla*_KPC_ gene is likely located within the pKpQI-IT-like plasmid in strain VA360. JHCK1 and VA360 genomes have 80 and 83 tRNA genes, respectively, as well as 4 rRNA genes.

Of all the predicted genes, 4,973 are common between the JHCK1 and VA360 genomes, although some of the other genes do have homologs in other sequenced genomes. Of the remaining 839 genes in JHCK1, 267 have an assigned function, including involvement in iron acquisition, conjugative transfer, and copper, arsenic, fosfomycin, mercury, and chromate resistance. Similarly, of the 551 VA360 genes not found in JHCK1, 175 have an assigned function, including those involved in monosaccharide metabolism, citrate metabolism/transport, and resistance to aminoglycosides (adenylyltransferase). These functions may be indicative of strain-specific capabilities.

### Nucleotide sequence accession numbers. 

The GenBank accession numbers for *K. pneumoniae* JHCK1 (Argentina) and VA360 (Cleveland, OH) are ANGH00000000 and ANGI00000000, respectively.
